# Enablers of and barriers to high quality clinical supervision among occupational therapists across Queensland in Australia: findings from a qualitative study

**DOI:** 10.1186/s12913-015-1085-8

**Published:** 2015-09-24

**Authors:** Priya Martin, Saravana Kumar, Lucylynn Lizarondo, Ans VanErp

**Affiliations:** Cunningham Centre, Darling Downs Hospital and Health Service, Queensland Health, Toowoomba Hospital, Private Mail Bag 2, Pechey Street, Toowoomba, QLD 4350 Australia; School of Health Sciences, International Centre for Allied Health Evidence, University of South Australia, GPO Box 2471, City East Campus, Adelaide, South Australia 5001 Australia; Joanna Briggs Institute, Wyatt House University of Adelaide, 115 Grenfell Street, Adelaide, South Australia 5000 Australia

**Keywords:** Clinical supervision, Occupational therapy, Qualitative methods

## Abstract

**Background:**

Health professionals practising in countries with dispersed populations such as Australia rely on clinical supervision for professional support. While there are directives and guidelines in place to govern clinical supervision, little is known about how it is actually conducted and what makes it effective. The purpose of this study was to explore the enablers of and barriers to high quality clinical supervision among occupational therapists across Queensland in Australia.

**Methods:**

This qualitative study took place as part of a broader project. Individual, in-depth, semi-structured interviews were conducted with occupational therapy supervisees in Queensland. The interviews explored the enablers of and barriers to high quality clinical supervision in this group. They further explored some findings from the initial quantitative study.

**Results:**

Content analysis of the interview data resulted in eight themes. These themes were broadly around the importance of the supervisory relationship, the impact of clinical supervision and the enablers of and barriers to high quality clinical supervision.

**Discussion:**

This study identified a number of factors that were perceived to be associated with high quality clinical supervision. Supervisor-supervisee matching and fit, supervisory relationship and availability of supervisor for support in between clinical supervision sessions appeared to be associated with perceptions of higher quality of clinical supervision received. Some face-to-face contact augmented with telesupervision was found to improve perceptions of the quality of clinical supervision received via telephone. Lastly, dual roles where clinical supervision and line management were provided by the same person were not considered desirable by supervisees. A number of enablers of and barriers to high quality clinical supervision were also identified.

**Conclusion:**

With clinical supervision gaining increasing prominence as part of organisational and professional governance, this study provides important lessons for successful and sustainable clinical supervision in practice contexts.

## Background

There are multiple challenges inherent in ensuring effective service delivery in highly dispersed populations such as those found in Queensland, Australia [1, 2]. Queensland is a geographically large state with a highly dispersed population of 4.5 million [3]. Public health services in Queensland are offered by 16 Hospital and Health Services where almost 5000 professionals from 16 discipline groups are employed [4]. Occupational therapy is one of the larger professions with services being provided across metropolitan, regional, rural and remote parts of the state. Health professionals including occupational therapists practising in non-metropolitan areas of the state often practise in professional isolation with the nearest professional colleague hundreds or thousands of kilometres away [5]. Clinical supervision (CS) is a key method of accessing professional support in such instances.

CS can sometimes be considered an ill-defined activity [6]. In this study, CS is defined as “the formal provision, by approved supervisors, of relationship-based education and training that is work-focused and which manages, supports, develops and evaluates the work of colleague/s” [7]. This type of supervision involves reflective thinking, discussion regarding professional development issues, caseloads, clinical issues, and staff interpersonal issues [8]. This is sometimes referred to as “supportive supervision” where there is joint problem-solving and two-way communication [6].

Within the Queensland public health service CS is widely recognised and encouraged. The credentialing and defining the scope of clinical practice Health Service Directive [9] and the associated Health Service Directive Guideline [10] provide direction and guidance to health professionals including occupational therapists regarding CS. Accordingly, newly graduated allied health professionals with less than two years full-time experience are expected to undertake one hour of formal CS per week; those with two to five years full time experience, a minimum of one hour per fortnight; and those with over five years experience, a minimum of one hour per month. While these requirements are made known to all allied health professionals, little is known about how CS is conducted or what makes it effective.

Most of the CS studies reported in the literature have focussed on nurses [11, 12]. A review of the CS literature has revealed key gaps such as a lack of agreement regarding methods of determining the quality of the CS process [13] and a lack of high quality studies investigating the quality and effectiveness of CS provided or received, especially among allied health professionals [11, 12, 14–16]. It has also been acknowledged in the literature that there is a lack of rigorous primary research studies into CS of non-metropolitan health staff in countries such as Australia [17].

### Benefits of clinical supervision

Historically, CS has been subjected to some criticism in the literature due to a lack of robust causal evidence linking it to improved clinical outcomes. For example, a systematic review of 18 studies that explored the impact of CS on counsellors and therapists, their practice and their clients found that links to improved client outcomes were only tentative [16]. Similarly, another systematic review of 25 empirical CS projects in psychiatric nursing found that only tentative conclusions were able to be made regarding the effect of CS [18].

Despite these limitations, there is some evidence for the effectiveness of CS. A study by Bambling et al. [19] of clients with major depression demonstrated that CS had a significant effect on a range of factors such as symptom reduction and treatment of clients. Furthermore, CS has been shown to benefit the practitioner, the patient and the organisation [20]. Reported benefits for practitioners include reduced isolation and burnout, improved coping at work and enhanced competence [21–24]. Reported benefits for patients include improved patient outcomes and better quality of care [25, 26]. Reported benefits of CS to the organisation include improved team work, improved clinical standards and enhanced quality of service delivery [19, 27]. Given the role CS plays in effective and high quality health care [19, 26] it warrants further investigation to determine what factors make it effective.

This qualitative study, with a specific focus on enablers of and barriers to high quality CS, was undertaken as part of a larger mixed methods study of the factors that contribute to high quality CS in occupational therapy across Queensland in Australia. The findings from the quantitative arm of this research will be reported in due course. The focus of this research was occupational therapists, as they are a well-established allied health profession and as CS forms an integral part of the localised governance framework.

## Methods

### Ethics

Ethics approval was obtained from the Metro South Hospital and Health Service Human Research Ethics Committee for multi-sites [Reference number: HREC/11/QPAH/322, Approval date: 6^th^ September 2011]. Following this, site specific approvals were obtained from 17 Hospital and Health Services in the state of Queensland.

### Design

This qualitative study was undertaken as part of a mixed methods sequential explanatory research study [28]. The qualitative study consisted of individual, in-depth, semi-structured interviews with supervisee occupational therapists.

### Setting

This study was conducted in the public health service organisation in Queensland comprising of 17 Hospital and Health Services (which have now been re-organised into 16 Hospital and Health Services) [4].

### Participants

As with any qualitative research, the purpose of sampling and sample size is not to represent the population. Sampling and sample size in qualitative research is influenced by methodological and practical considerations. Practical considerations include time, costs and resources required to undertake data collection and analysis. Methodological considerations include data saturation and variability within the target population. Taking these into account, participants for this research were recruited using a purposive maximum variation sampling strategy [29]. This sampling strategy captures diverse variations and helps identify common patterns that cut across variations [29]. Directors of occupational therapy and clinical education support officers across metropolitan, regional, and rural Hospital and Health Services were contacted and requested to nominate potential participants for the interviews. They were particularly asked to nominate staff from a broad range of experience and nature of role to capture diverse perspectives. Staff nominated had to be occupational therapists employed with Queensland Health and currently receiving CS. Those nominated were then contacted to obtain informed consent for the interviews.

### Procedure

Nine individual, in-depth, semi-structured interviews were completed. The interviewees generally characterised the target population. In addition to practical considerations like cost and time, it became apparent that at the end of nine interviews no new information was forthcoming and the concepts emerging became similar and repetitive making any further data collection redundant [30].

An interview guide was developed based on the results from the quantitative study and literature findings. The quantitative study consisted of a survey of 207 supervisee occupational therapists in Queensland. A demographic questionnaire and the Manchester Clinical Supervision Scale were completed by the participants. Information about what factors influenced the quality of CS were obtained. Participants also had the opportunity to include free text comments about their CS experience in general.

The interview guide consisted of a comprehensive set of open-ended questions and additional relevant prompts. The purpose of the guided interviews were to elicit the participant’s worldview. As per this method, the researcher identified a few broad topics (framed as questions) to help uncover the participant’s meaning or perspective but otherwise respected how the participants framed and structured responses. The researcher posed open-ended questions followed by requests for elaboration [31]. Some of the categories and questions included in the interview guide were on supervisory relationship, enablers of and barriers to CS, dual relationships and telesupervision. Appendix A lists the categories and questions that were included in the interview guide. All interviews were conducted via telephone.

### Data analysis

The interview data were recorded with permission, transcribed verbatim and subject to inductive content analysis. The approach while using inductive content analysis was to move from the specific to the general to observe particular instances and combine them into a larger whole [32]. Qualitative content analysis and thematic analysis are two analysis approaches frequently used in qualitative descriptive approaches [33]. Although both content and thematic analyses cut across data and search for patterns and themes, content analysis provides the opportunity for quantification of data [33]. Content analysis also allows the researcher to measure the frequency of different categories and themes [33] and hence was chosen as the preferred method of analysis.

A range of techniques were employed to enhance the research rigour of the qualitative data analysis and interpretation processes. Strategies used to promote credibility, transferability, dependability and confirmability of the data analysis and interpretation processes included adherence to the semi-structured interview guide, audiotaping interviews, transcribing verbatim by an independent typist, coding by more than one investigator, cross checking between coders and member checking of a proportion of the data [31, 34–36]. Data were de-identified to promote trustworthiness of the analysis process. To avoid conflicts of interest, threats to accuracy of data and to maintain confidentiality, a potential interview participant that was nominated was excluded as that participant was in a previous CS partnership with the principal investigator.

## Results

The interview participants were from work places that represented a range of geographical areas in Queensland such as metropolitan, regional, rural and remote. Position classification of the participants and experience levels in the profession and the role also varied broadly. Participants reported receiving one-to-one CS, peer group supervision and/or mentoring in their current roles. Further demographic details of the participants have been included as Table 1. Content analysis of the interview data resulted in the following eight themes:

**Table 1:** Participant demographics and information about CS received (*n* = 9)

Demographic characteristics	Number
Gender	
Male	0
Female	9
Workplace Location	
Metropolitan	3
Regional	2
Rural	2
Remote	2
Nature of role	
Clinical	8
Non-clinical	1
Position Classification	
Health Practitioner level 3	5
Health Practitioner level 4	2
Health Practitioner level 5	2
Mode of CS	
Face-to-face	3
Telephone	6
Relationship with the supervisor	
Knew supervisor beforehand	4
Did not know the supervisor beforehand	3
Choice of supervisor	
Chose their supervisor	4
Supervisor was allocated	5

### Supervisor-supervisee matching/fit is important

A predominant theme arising from the interviews was that supervisee-supervisor matching and fit were important. When the supervisee was matched carefully with a suitable supervisor, the supervisory relationship was reported to be more positive and effective. Being provided with a choice of supervisor was considered desirable by most participants. One supervisee commented thus:

“I am actually really happy with the supervision I am getting at the moment. I was very, very lucky to get the supervisor I have right now. I proactively sourced my supervisor in order to get somebody who was suited to not only my background but also my clinical level, so yeah, I’m really pleased with it”

### Supervisory relationship affects supervisee perceptions of the effectiveness of CS

When the supervisory relationship was reported to be positive and supportive, CS was considered to be effective. All participants that reported a positive supervisory relationship also reported satisfaction with their CS arrangements. One participant described the supervisory relationship with her supervisor as supportive, considerate, understanding and positive. When this participant was asked about the ways in which such a supervisory relationship affected her supervision she said:

“It affects it in a positive way, like you definitely make the time to have that regular supervision and you make the time to sit down and obviously do things as a result of your supervision. So yeah, it leaves you with a more positive work environment for yourself”

### Availability of supervisor between CS sessions enhances the perceived effectiveness of CS

Many participants considered having access to the supervisor between CS sessions as desirable. Those that reported having access to their supervisors and felt comfortable contacting them in between sessions when required, reported their CS to be effective. One participant remarked:

“…It’s handy to have the other person (supervisor) there. I email her in between times to ask questions…”

### Validation is an important function of CS

Participants generally regarded validation as an important function of CS. They reported that CS provides them with a chance to hear someone else’s perspective and obtain feedback on their practise. One participant said:

“….I actually really enjoy it (CS)…. I find it really affirmative…. I think probably the sense of security I get with having that relationship is what I value the most”.

### There is a general sense of confusion regarding CS

Many participants appeared to be confused about what CS entailed. Confusion was especially noted around terminologies related to supervision. Some participants found it difficult to differentiate CS from peer group supervision and mentoring. Few participants reported receiving more than one type of supervision and were unable to differentiate one form the other. One participant said:

“….to be really honest, I think it’s more about clarification of what you mean by supervision…because there is supervision and there is supervision. There is supervision with constructive feedback , there is supervision with you know actually assisting you to know as a clinician and challenge your thoughts….and there is supervision to make sure your mandatory training is up to date….”

### Dual roles are not considered optimal

All participants agreed that they would like to keep their CS separate from line management. When asked about CS and line management being offered by the same person, one participant said:

“I don’t think I would like that arrangement because sometimes you might want to discuss things, you know, professional issues you have, that may involve your line manager or you want advice on how to tackle that or talk about that to your line manager and that would be pretty hard when your line manager is your supervisor”

Another participant that was receiving CS from her team leader/manager said:

“Supervision is affected by other points I mentioned before because it is not 100 % focused on me and my goals, it is often focused on the department goals or her goals”

### Some face-to-face contact is considered to make CS via telephone more effective

There was general consensus that adjunct face-to-face contact with the supervisor or prior face-to-face interaction with the supervisor even if outside the supervision context, made CS received over the telephone more effective.

A participant that was receiving CS from a supervisor she had never met face-to-face expressed numerous concerns about the effectiveness of CS received and the supervisory relationship. She said:

“…I think it is really difficult, especially when you are starting to get to know someone…yeah it’s really tricky because you don’t have any of that, you know, body language or you know facial expressions to sort of get some feedback on what you are saying and I think that is difficult…just not being able to see someone it’s difficult, probably more difficult to develop rapport”

On the other hand, another participant that received CS from a supervisor known to her from before entering the CS partnership stated:

“… It does help that I have met her face-to-face, you know that makes a huge difference to me…I don’t think I could do it as well if I hadn’t met the person face-to-face…”

### Enablers of and barriers to effective CS

Participants were asked about the different enablers of and barriers to CS they encountered. Enablers of effective CS reported include face-to-face mode of supervision, good supervisor-supervisee fit, CS sessions being supervisee-driven, structured sessions, use of agendas, having an action plan for follow-up after sessions, being organised, having clear expectations, having flexibility, and open communication with the supervisor. Some of the barriers to effective CS reported include lack of time, other workload commitments, being located in a non-metropolitan area, dual roles, supervisor being unavailable and unwelcoming, unstructured sessions, lack of confidential space to hold sessions, having a different learning style to that of the supervisor and having a negative supervision culture in their work unit where CS is not valued.

When asked about the facilitators of effective CS, one participant reflected on a previous CS partnership that she described as the ‘best’ supervision experience she had so far. She said:

“… my supervisor and I had the same learning styles, we were both reflective learners, we set clear goals at the very start of supervision…. Everything was very structured…we had lots of debriefs….. my supervisor was always available to talk about the challenges on the ward….at the end of the rotation, I could see where I had started and where I had finished and what I had achieved… it was very neat and effective”

Another participant while commenting on barriers to effective CS said:

“…one of the critical things is the barrier when people aren’t clear about what they want, and what they get supervision for… so it becomes a catch up coffee shop stop and I do lots of supervision in a coffee shop but that doesn’t mean you don’t have a clear vision of what your agenda is and why you are doing it”

## Discussion

There is a growing body of evidence on the impact of CS across a range of health professions [20] and the important role CS can play, including in non-metropolitan settings [36]. As this research spans across metropolitan and non-metropolitan regions, it provides insight into the factors that contribute to high quality CS across a range of geographical areas. As CS has been under-researched in some allied health professions, such as occupational therapy, findings from this study are expected to contribute to the evidence-base in this area.

Findings pertaining to the importance of supervisor-supervisee matching and fit concur with previous research findings by Ducat et al. [36]. Findings from that multi-disciplinary study highlighted that a good supervisor-supervisee fit was of critical importance. Results indicated that a poor match often resulted in ineffective CS. The participants in that study reported that a good supervisor-supervisee fit resulted in a positive relationship that was reciprocal [36]. A good supervisor-supervisee fit influences the supervisory relationship which has been identified as the single most important factor for effective CS in many empirical studies [14, 37, 38]. Findings from this study reinforce the importance of the supervisory relationship as those participants that reported positive, supportive relationships also reported that their CS was effective.

Dual roles were not considered desirable by most participants in this study. When CS and operational or line management are provided by the same person, there is a risk of departmental issues taking priority over the supervisee’s learning needs. This is consistent with an allied health study by Dawson et al. [39] which reported that supervisees’ expressed desire to better separate line management and CS. It appears that CS is more effective when provided by someone other than the supervisee’s manager. This is because such an arrangement promotes a confidential environment for the supervisee where they are better able to disclose information and discuss issues of concern without ‘guarding’. It is also likely that if CS and operational or line management were provided by the same person, due to clinical priorities and competing work demands, CS may be seen a “poor cousin” in the work context and hence pushed down the list of priorities.

A number of participants in this study received CS via telephone. There was general consensus that this arrangement worked better when they had some prior interaction with the supervisor (before entering the CS partnership) or had met the supervisor face-to-face. CS via telephone denies the supervisee access to the supervisor’s non-verbal communication. The effect of this appears to be more detrimental when the supervisee has not had prior face-to-face interactions with the supervisor. These findings highlight supervisees’ need for supplemented face-to-face contact while receiving CS via telephone. This is consistent with the telehealth model for CS proposed by Wood, Miller and Hargrove [40]. As per this model, telesupervision supplemented with on-site face-to-face CS is considered helpful for trainees and new practitioners. However, it must be acknowledged that it is not always possible for rural and remote health practitioners to have supplementary face-to-face CS to adjunct telesupervision. This is due to barriers such as geographical issues, time, resources available for travel and competing workload and clinical priorities. Despite this, as many health professionals, especially in rural and remote settings, use technology to receive CS, ongoing further research is warranted to explore the uptake of and the impact from the use of technology to receive CS.

### Limitations

Despite generating new knowledge about important issues pertinent to CS in occupational therapy, there are some limitations to this research. This research was conducted with nine participants, all female, from one geographical location in Australia (Queensland). While this may be considered as a limitation as the findings cannot be generalised to the broader population of occupational therapists, they nevertheless provide some useful insight into CS issues that require ongoing exploration and research. While the qualitative arm of the study explored the quality of CS from a supervisee’s perspective, it did not explore the supervisor’s perspective. Further studies are required to explore this. Whilst this study provides rich information about CS in occupational therapy, it does not provide a multidisciplinary perspective. Further research is required especially in a range of allied health professions to investigate what factors make CS effective.

## Conclusions

Health professionals such as occupational therapists practising in highly dispersed geographical locations such as in Queensland, Australia rely on CS for professional support and guidance. While policies and guidelines exist in organisations such as Queensland Health regarding minimum CS requirements, little was known about how CS was actually conducted and what factors impacted the quality of CS received by supervisees. This study explored the enablers of and barriers to high quality and effective CS of occupational therapists in Queensland from a supervisee perspective.

The findings from this study contribute to the growing evidence base for CS in allied health. The findings from this study indicate that a number of factors were perceived to be associated with high quality CS. Supervisor-supervisee matching and fit, supervisory relationship and availability of supervisor for support in between CS sessions appeared to be associated with perceptions of higher quality of CS received. Some face-to-face contact appeared to improve perceptions of the quality of CS received via telephone. Supporting previous literature findings, the findings from this study also highlight a general sense of confusion regarding supervision and the associated terminology. Lastly, dual roles, where someone received CS and line management from the same person, were not considered desirable. A number of enablers of and barriers to high quality CS have also been identified. As CS gains prominence as part of organisational and professional governance, it is imperative that implementing best practice CS is underpinned by current best evidence regarding “what works” in practice contexts. The findings from this study provide important lessons for successful and sustainable CS implementation in practice contexts.

## Appendix A

### Interview guide

Some of the categories and questions included in the interview guide were:

- Clinical supervision (CS)
  - Tell me about the CS you receive
  - How do you find the CS you receive?
  - Is your supervisor also your line manager?
  - What would you like to say about dual relationships where a supervisee’s clinical supervisor is also their line manager?
- Supervisory relationship
  - Tell me about your relationship with your supervisor?
  - How does this relationship affect your CS?
- Facilitators of and barriers to effective CS
  - Let us think about what you would ideally like your CS to be like. What would you like it do for you?
  - If you could make your CS exactly how you would like it to be, what would it be like? What would make it more effective?
  - Think about the ideal things that you just talked about. Are there things that get in the way of achieving that type of CS?
- Telesupervision
  - Do you receive CS via telephone?
  - How do you find the CS you receive via telephone?
  - Are there things that make the telesupervision arrangement less effective for you?

## Abbreviations

CS: Clinical supervision
